# Lipid Metabolism, Carcass Characteristics and *Longissimus dorsi* Muscle Fatty Acid Composition of Tropical Crossbred Beef Cattle in Response to *Desmanthus* spp. Forage Backgrounding

**DOI:** 10.3390/metabo11120804

**Published:** 2021-11-27

**Authors:** Felista W. Mwangi, David J. C. Blignaut, Edward Charmley, Christopher P. Gardiner, Bunmi S. Malau-Aduli, Robert T. Kinobe, Aduli E. O. Malau-Aduli

**Affiliations:** 1 Animal Genetics and Nutrition, Veterinary Sciences Discipline, College of Public Health, Medical and Veterinary Sciences, Division of Tropical Health and Medicine, James Cook University, Townsville, QLD 4811, Australia; felista.mwangi@my.jcu.edu.au (F.W.M.); david.blignaut@jcu.edu.au (D.J.C.B.); christopher.gardiner@jcu.edu.au (C.P.G.); robert.kinobe@jcu.edu.au (R.T.K.); 2 CSIRO Agriculture and Food, Private Mail Bag Aitkenvale, Australian Tropical Sciences and Innovation Precinct, James Cook University, Townsville, QLD 4811, Australia; Ed.Charmley@csiro.au; 3 College of Medicine and Dentistry, Division of Tropical Health and Medicine, James Cook University, Townsville, QLD 4811, Australia; bunmi.malauaduli@jcu.edu.au

**Keywords:** carcass traits, meat quality, intramuscular fat, fat melting point, fatty acids, tropical beef cattle

## Abstract

Lipid metabolism, carcass characteristics and fatty acid (FA) composition of the *Longissimus dorsi* (loin eye) muscle were evaluated in tropical crossbred steers backgrounded on *Desmanthus* spp. (desmanthus) with or without feedlot finishing. It was hypothesized that steers backgrounded on isonitrogenous diets augmented with incremental proportions of desmanthus will produce carcasses with similar characteristics and FA composition. Forty-eight Brahman, Charbray and Droughtmaster crossbred beef steers were backgrounded for 140 days on Rhodes grass (*Chloris gayana*) hay augmented with 0, 15, 30 or 45 percent desmanthus on dry matter basis. Lucerne (*Medicago sativa*) hay was added to the 0, 15 and 30 percent desmanthus diets to ensure that they were isonitrogenous with the 45 percent desmanthus diet. After backgrounding, the two heaviest steers in each pen were slaughtered and the rest were finished in the feedlot for 95 days before slaughter. Muscle biopsy samples were taken at the beginning and end of the backgrounding phase. Carcasses were sampled at slaughter for intramuscular fat (IMF) content, fat melting point (FMP) and FA composition analyses. Increasing the proportion of desmanthus in the diet led to a linear increase in docosanoic acid (*p* = 0.04) and omega-6/omega-3 polyunsaturated FA ratio (n-6/n-3 PUFA; *p* = 0.01), while docosahexaenoic acid decreased linearly (*p* = 0.01). Feedlot finishing increased hot carcass weight, subcutaneous fat depth at the P8 site and dressing percentage (*p* ≤ 0.04). The n-6/n-3 PUFA ratio was within the recommended < 5 for human diets. IMF was within the consumer-preferred ≥3% level for palatability. The hypothesis that steers backgrounded on isonitrogenous diets augmented with incremental proportions of desmanthus will produce similar carcass characteristics and FA composition was accepted. These findings indicate that a combination of tropical beef cattle backgrounding on desmanthus augmented forage and short-term feedlot finishing produces healthy and highly palatable meat.

## 1. Introduction

Beef is the third most consumed meat in the world at 14.4 kg per capita after poultry and pork at 33.0 and 22.9 kg per capita, respectively [1]. World total meat production in 2020 from ovine, bovine, poultry, pig and other animals was estimated at 337.2 million tonnes carcass weight equivalent, 62% of which was produced in Brazil, Australia, USA, EU, China, India and Argentina [2]. Therefore, meat is a significant global source of high quality protein, dietary lipids, minerals and B vitamins [3]. As a result of the 2016 controversial epidemiological suggestion of red meat consumption being linked to increased risks of cancer, cardiovascular disease, obesity and diabetes by Troy et al. [4], recommendations by the American Heart Association [5]) and the World Health Organization [6] to reduce red meat consumption, have been vigorously challenged by the Nutritional Recommendations Consortium [7]. Saturated fatty acids (SFA) have been reported to increase plasma low density lipoprotein cholesterol levels [8], while other researchers reported an inverse relationship between SFA intake and the incidence of stroke [9]. In some population-based studies [10], the dietary intake of conjugated linoleic acid (CLA) has been inversely linked to the risk of breast cancer and colorectal cancer. In contrast, prospective cohort studies consistently support the role of eicosapentaenoic acid (EPA) and docosahexaenoic acid (DHA) in the primary prevention of atherosclerosis and cardiovascular disease [11] and a reduction in the omega-6/omega-3 (n-6/n-3) ratio known to be associated with highly reduced risk of obesity [12]. In addition to health benefits, carcass fat influences meat quality and palatability by influencing meat tenderness, shelf-life, juiciness, flavour and market value [13].

Diet has a significant effect on meat quality [14]. Whereas numerous studies exist on increasing meat omega-3 polyunsaturated fatty acids (n-3 PUFA) composition through dietary supplementation with fish oil, fish meal and vegetable oils [15,16,17], similar cattle supplementation studies in the extensive grazing systems of northern Australia are scanty, cost being a significant limiting factor [18]. Forages contain a high proportion of total FA as alpha-linolenic acid (ALA) which is the building block of the longer chain (≥C20) n-3 PUFA through elongation and desaturation [19,20]. Plant secondary metabolites such as tannins and saponins may modify meat FA composition by modulating rumen FA lipolysis and biohydrogenation [21,22]. For instance, the intramuscular fat (IMF) of lambs fed tannin-containing birdsfoot trefoil (*Lotus corniculatus*) and sainfoin (*Onobrychis viciifolia*) were shown to contain less SFA and more PUFA compared to those fed lucerne or red clover [17] and supplementing growing lambs with lucerne saponins at 0.05–0.2% DM for 90 days significantly increased muscle n-3 PUFA [23]. These findings necessitate the need to study the effect of supplementing beef cattle with forage of the genus *Desmanthus* (desmanthus)*,* a tannin-containing and vigorous tropical legume that is environmentally well-adapted to the harsh tropical and subtropical conditions of northern Australia [24], on meat fatty acid (FA) composition. Desmanthus is a highly productive forage legume that thrives with as low as 500 mm annual rainfall, withstands high grazing pressure and has been shown to reduce enteric methane emission in cattle fed low quality grass diet [25,26].

Backgrounding (also referred to as stocker phase) cattle prior to feedlot finishing with energy-dense diets is commonly practiced in order to meet the carcass specifications of premium beef markets. Approximately half of the beef cattle herd produced in northern Australia [27], Mediterranean countries [28] and 85% in the USA are finished in the feedlot [29] for improved subcutaneous fat depth [28] and IMF content that markedly increase flavour, tenderness and juiciness [19]. By the same token, finishing cattle on concentrate diets also has a tendency to increase meat SFA and reduce the health beneficial unsaturated FA concentration [30,31]. To our knowledge, no peer-reviewed literature exists on the intramuscular FA composition of tropical crossbred beef cattle backgrounded on desmanthus forage. Therefore, this study aimed to fill this knowledge gap by evaluating the feedlot performance, carcass characteristics, IMF, FMP (fat melting point) and FA composition of the *Longissimus dorsi* (loin eye) muscle of tropical crossbred beef steers backgrounded on desmanthus forage with or without feedlot finishing. It was hypothesised that steers backgrounded on isonitrogenous diets augmented with incremental proportions of desmanthus will produce similar carcass characteristics and FA composition.

## 2. Results

### 2.1. IMF, FMP and FA Composition 

The backgrounding diet did not have a significant influence on the loin eye muscle IMF content, FMP and FA composition (*p* ≥ 0.20), with the exception of C22:0 (Docosanoic acid) (*p* = 0.04) and n-6/n-3 PUFA ratio (*p* = 0.01) that increased linearly with an increase in the proportion of desmanthus in the diet (Table 1). The steers were lean, with IMF content ranging between 2.1% and 2.6% (*p* = 0.50) and FMP from 43.1 °C to 44.5 °C (*p* = 0.94). Although the desmanthus inclusion numerically increased total FA content, this increase did not reach statistical significance (*p* = 0.59). 

**Figure 1:**
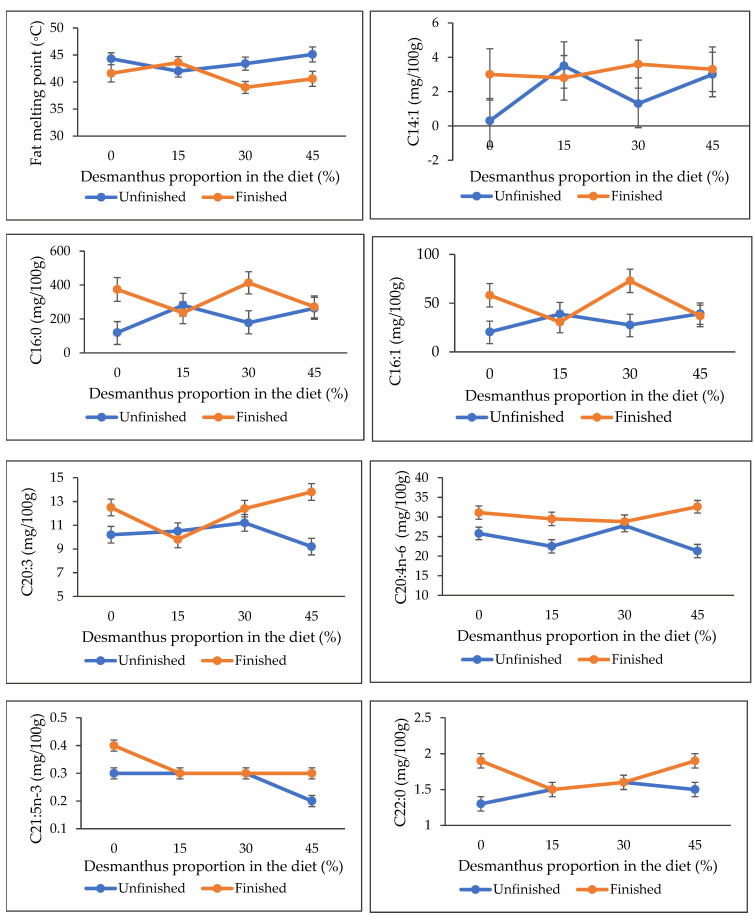
Effect of diet and feedlot finishing interactions on fat melting point (FMP), C14:1, C16:0, C16:1, C20:3, C20:4n-6, C21:5n-3 and C22:0 fatty acids composition (*p* ≤ 0.04).

The IMF, FMP and FA composition data of forage backgrounded steers with or without feedlot finishing are presented in Table 2. No effect of backgrounding diet was observed on the IMF, FMP and FA composition (*p* ≥ 0.14) except for the DHA levels that linearly declined with an increase in desmanthus proportion in the diet (*p* = 0.01). In contrast, feedlot finishing of the steers increased the IMF, oleic acid, CLA, LA, ARA, ∑MUFA, ∑n-6 PUFA and n-6/n-3 PUFA levels and reduced ALA and ∑n-3 PUFA (*p* ≤ 0.04). Feedlot finishing increased DPA (*p* = 0.04) and ∑SFA (*p* = 0.02) except in steers fed 30% and 15% desmanthus diets, respectively, while PUFA/SFA ratios decreased for the steers fed the 0 and 30% (*p* = 0.04) but remained unchanged for the 15% and 45% desmanthus diets steers. No significant effect of feedlot finishing was observed on the EPA, DHA, EPA+DHA or EPA+DPA+DHA levels (*p* ≥ 0.16). Interactions between diet and finishing were observed for the FMP, C14:1, C16:0 (palmitic acid), C16:1, C20:3, ARA, C21:5n-3 and C22:0 (Figure 1; *p* ≤ 0.04). The FMP was lower for the feedlot finished steers in all diets except the 15% desmanthus diet, and higher for the feedlot finished than the unfinished steers at 43.6 and 42.0 °C, respectively. A reverse trend was observed for palmitic acid, where it was lower for the feedlot finished than the unfinished steers at 236.5 mg/100 g and 280.6 mg/100 g muscle, respectively.

### 2.2. Feedlot Growth Performance and Carcass Characteristics 

**Figure 2:**
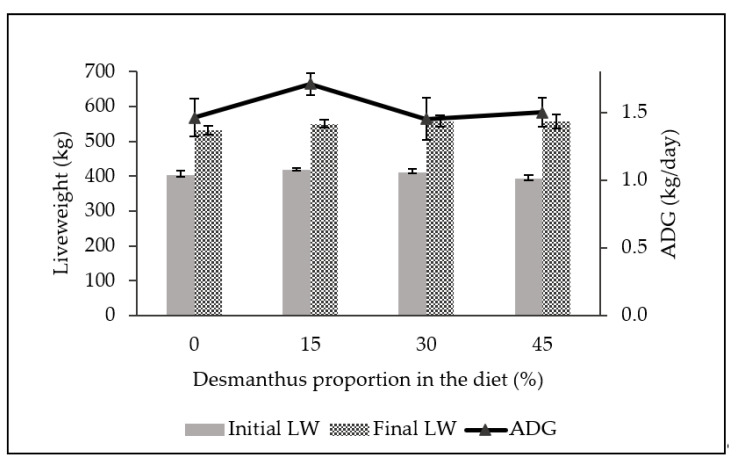
Mean (±SD) initial liveweight (initial LW; *p* = 0.36), final liveweight (final LW; *p* = 0.63) and average daily gain (ADG; *p* = 0.41) of steers during feedlot finishing after backgrounding on diets with incremental desmanthus.

The initial liveweight (LW), final LW and average daily gain (ADG) of steers ranged between 330.2–332.8 kg, 420.1–437.8 kg and 0.53–0.62 kg/day, respectively, during the finishing phase (Figure 2). There was no significant difference in growth performance of steers backgrounded on either diets (*p* ≥ 0.36).

The slaughter LW and carcass characteristics of backgrounded steers with or without feedlot finishing are presented in Table 3. Feedlot finished steers had higher slaughter weight, subcutaneous fat depth at the P8 site, hot carcass weight (HCW) and dressing percentage (*p* ≤ 0.04), but no difference was observed between diets (*p* ≥ 0.28).

## 3. Discussion

### 3.1. Intramuscular Fat Content, Fat Melting Point and Fatty Acids Composition

Steers backgrounded on diets augmented with incremental proportions of desmanthus or lucerne had similar IMF, FMP and FA composition. These results agree with results reported in previous studies. For instance, Dierking et al. [32] reported similar FA composition in the loin eye muscle of steers finished on tall fescue (*Lollium arundinaceum*) combined with either red clover (*Trifolium pretense L*.) or lucerne pastures. Beef steers offered grass silage mixed with lupins/triticale silage or vetch/barley silage for 122 days had similar IMF content and FA composition in the loin eye muscle [33]. A study of steers finished on lucerne or mixed pasture consisting of bluegrass (*Poapratensis* L.), orchardgrass (*Dactylis glomerata* L.), tall fescue (*Festuca* L.), and white clover (*Trifolium repens L*.) reported similar IMF and FA composition, except for ALA that was higher for the lucerne than the mixed pasture finished steers [34]. Steers fed birdsfoot trefoil or meadow brome (*Bromus riparius*) grass had similar IMF, SFA, MUFA, PUFA, PUFA/SFA ratio and n-6/n-3 ratio, although EPA was higher for the birdsfoot trefoil [35]. In this study, steers were lean with IMF of 2.1–2.3% after backgrounding. IMF accumulation depends on the balance between uptake, synthesis and degradation of triacylglycerols, hence increasing the availability of net energy for fat synthesis during finishing results in higher IMF content [19]. Backgrounding diets had low ME content (7.4–8.1 MJ/kg DM), hence the low IMF content was expected. The increase in IMF, FMP and SFA after feedlot finishing steers agrees with previous studies [19,36,37]. IMF is positively correlated with carcass fatness and an increase in carcass fatness is reported to improve meat tenderness through the insulation of the subcutaneous and intermuscular fat against the effect of refrigeration during carcass cooling and reduction of the resistance to shearing through the accumulation of IMF in the perimysial connective tissue [38]. IMF is negatively correlated with drip loss and cooking loss, indicating that increasing IMF may improve meat water-holding capacity and consequently higher juiciness score [37]. Hence a minimum of 3% IMF is required to meet consumer-preferred overall palatability [39]. The 3% IMF was achieved in all the carcasses of feedlot finished steers but only in the 0 desmanthus diet for the unfinished steers indicating that finishing desmanthus backgrounded steers is necessary to meet the consumer-preferred palatability score.

All the melting points were within the 30–50 °C range reported for beef cattle IMF [40,41]. FMP is influenced by the melting points of its FA components. For instance, stearic acid (C18:0) melts at 69.6 °C, whereas oleic acid (C18:1) melts at 13.4 °C hence the concentration of stearic acid in beef fat has the greatest effect on FMP [42]. Stearic acid and palmitic acid are among the dominant FA in beef fat and both are reported to increase the fat hardness [39] and subsequently the FMP [42]. In this study, there was no difference in stearic acid concentration between the steers backgrounded on the different diets hence the similar FMP. However, the decrease in FMP after finishing may be due to an increase in carcass fatness as indicated by increased IMF [41]. Wood et al. [13] reported that fat cattle have soft and oily fat due to increased oleic relative to stearic acid. The firmness of fat influences economics of meat processing and the overall consumer acceptance [42], with softer fat preferred because it is easier to process during carcass boning and improves meat flavour [43]. Besides, increased muscle oleic acid is associated with greater beef palatability, linked to the softness that provides a more fluid mouthfeel [39,44]. Oleic acid is reported to lower blood low density lipoprotein and may increase the high-density lipoprotein making it a heart-healthy dietary fat [39]. Our findings of similar oleic acid concentration and FMP may indicate that the increasing proportion of desmanthus did not negatively impact carcass processing ease.

Plant metabolites such as polyphenol oxidase present in red clover are reported to reduce the activity of plant lipases [45,46], while tannins may reduce lipid biohydrogenation in the rumen, although tannins ability to inhibit biohydrogenation remains controversial [47]. A tannin-containing forage (Sainfoin) was reported to have no effect on ALA biohydrogenation. However, tannin extracts-containing diets (7.9% of dietary DM) reduced biohydrogenation by 20% in vitro [48], and the addition of sainfoin in a grass silage diet increased the accumulation of ALA in the rumen digesta of lambs [49]. A recent study reported that supplementing growing lambs with lucerne saponins at 0.05–0.2% DM for 90 days significantly increased muscle n-3 PUFA [23], although other studies reported that saponins had no effect on muscle n-3 PUFA, but it increased n-6 PUFA [47]. Lucerne saponin levels of 0.8% to 2% are common [50]. The lack of difference in the muscle SFA and unsaturated FA concentration of steers backgrounded on the different diets in this study may be due to the presence of plant secondary metabolites in both legumes. Feedlot finishing steers reduced the ALA concentration by over 50% compared with the unfinished steers. This outcome was anticipated due to the high concentration of ALA in forage [13]. Studies have reported that feeding ruminants with forage compared to concentrates with no added n-3 source results in higher concentrations of the health beneficial ALA, EPA, DPA and DHA in muscle lipids due to high concentration of ALA (50–75% of the total FA) in forage [19,51,52]. ALA is the building block of the n-3 PUFA, and its elongation and desaturation result in the synthesis of EPA and DHA [20]. Finishing steers resulted in a decline in ALA, DPA and total n-3 PUFA but EPA and DHA remained similar in this study. Concentrate feeding without n-3 supplementation reduces muscle n-3 PUFA levels due to the low ALA levels in concentrate diets [52,53]. The linear decline in DHA concentration with an increase in the proportion of desmanthus in the diet was not expected since the dietary ALA concentration was higher in desmanthus (34.9%) than in lucerne (26.5%) and Rhodes grass (25.6%). The difference could have been due to increased rumen biohydrogenation of ALA with an increase in dietary desmanthus proportion or an effect of desmanthus on elongase and desaturase enzymes. Diet modifies meat FA composition by influencing the expression of genes associated with FA synthesis and metabolism [54,55,56]. Therefore, more studies are required to examine the effect of desmanthus supplementation on the expression of lipogenic genes and examine if an interaction between lipogenic gene polymorphisms and the proportion of desmanthus in the diet exists.

The muscle fat level is inversely related to the PUFA/SFA ratio. As muscle fat increases, SFA and MUFA increase faster than PUFA, resulting in a decline in the relative proportion of PUFA, and consequently, in the PUFA/SFA ratio. Reviewing 24 studies, De Smet et al. [57] reported a strong inverse relationship between IMF and PUFA/SFA ratio in beef. IMF increased from 1 to 4% and PUFA/SFA ratio decreased from 0.7 to 0.1 (R^2^ = 0.85). The majority of muscle PUFA is found in the phospholipids and only a small amount is present in the triacylglycerols. The PUFA/SFA ratio is mainly influenced by genetics and overall carcass fat level and much less by nutrition. The PUFA proportion of phospholipids is strictly controlled to maintain membrane properties, whereas the PUFA in triacylglycerols is strongly linked to the total fat content and is reported to vary from 0.2 to 5% [57,58]. PUFA/SFA ratio for the forage fed and feedlot finished steers (0.3–0.6) in this study were similar to those reported by Aldai et al. [59] (0.4–0.7) and close or within the desired dietary ratio of 0.4 [60]. 

Similar to our findings, German Holstein and Simmental bulls fed forage-based diets had higher n-3 PUFA and lower n-6/n-3 ratios than their concentrate fed counterparts, although SFA levels were similar [61]. On the average, beef from pasture-fed steers contains higher concentrations of C20:3n-3, total n-3 PUFA and lower n-6/n-3 ratio in the IMF relative to those finished in the feedlot [36]. Feeding steers with concentrates for two months before slaughter after forage feeding resulted in a decrease in n-3 PUFA and an increase in n-6 PUFA concentration in the muscle [59]. In another study, pasture-fed cattle had lower SFA, MUFA and n-6/n-3 PUFA ratio, higher n-3 PUFA and similar PUFA levels compared to grain-fed cattle [62]. French et al. [58] also reported a lower n-6/n-3 PUFA in grass fed than concentrate fed cattle and they associated the difference with the higher dietary n-3 PUFA levels in the grass compared to the concentrate diet. Dietary guidelines recommend that the n-6/n-3 PUFA ratio for human diets should be below 5.0 [63], and ratios below 4.0 are purported to have a potential to decrease the risk of coronary diseases and cancer, while ratios of 1.0 or 2.0 may contribute to the prevention of obesity [12]. The n-6/n-3 ratios in this study (1.5–3.5) were all below 5.0 and similar or close to those reported for British cattle (2.0 to 2.3) [64], German Simmental bulls and Holstein steers (1.3) [65] and Alentejano cattle (1.8) [45]. Our findings indicate that backgrounding beef cattle on desmanthus augmented forage and finishing them in the feedlot for a short period (95 days) produces meat with a healthy n-6/n-3 PUFA ratio.

Increasing the proportion of desmanthus in the diet led to an increase in docosanoic acid concentration. These findings were contrary to that reported in a study where goats fed a basal diet of lucerne and augmented with Bermudagrass hay (*Cynodon dactylon*), Sericea lespedeza (*Lespedeza cuneate*) or pine bark (*Pinus taeda L.)* had similar docosanoic acid concentration in the loin eye muscle IMF [66]. Docosanoic acid is a long chain saturated FA found in trace levels in beef [67] from absorption of dietary sources [68] or produced directly from the biohydrogenation of DHA [69] or elongation of stearic acid [70]. Sheep fed grain pellets fortified with omega-3 oil had increased docosanoic acid concentration in the heart, liver and kidney, but not in the loin eye muscle [71]. Since the docosanoic acid concentration was similar between lucerne and desmanthus forages in this present study, increasing the proportion of desmanthus in the diet may have influenced docosanoic acid production through DHA biohydrogenation or stearic acid elongation. Although docosanoic acid is reported to increase serum cholesterol levels [68], its low proportion and absorption in the intestines, low bioavailability [72] and low concentration in the muscle [67] does not pose any detrimental impact on human health.

### 3.2. Feedlot Growth Performance and Carcass Characteristics 

Steers backgrounded on the different diets had similar weight gains during the feedlot finishing phase. The similar performance could be explained by the similar plane of nutrition (11.4–11.6 CP and 7.4–8.1 ME/kg DM) and ADG (0.52–0.66 kg/day) of all steers during the backgrounding phase echoed by the similar plasma non-esterified FA, glucose and β-hydroxybutyrate observed in our previous study (currently under review). Steen et al. [53] reported that heifers with low weight gains two months prior to the finishing phase had higher weight gains during finishing compared to steers who had higher weight gains prior. The authors associated the difference with compensatory growth in heifers, which was not the case in this study. The similar final LW between diets was expected due to similar initial weight and weight gain during finishing [34]. 

It has been reported that the carcasses of cattle grazing on tall fescue mixed with sainfoin or lucerne pastures had similar weights and subcutaneous rib fat depths [73] in agreement with this current study where no significant differences were observed. Other studies have similarly demonstrated that cattle backgrounded on different pastures (bermudagrass, indiangrass or a mixture of indiangrass, big bluestem and little bluestem) with similar LW at the end of backgrounding had similar LW, carcass weight, dressing percentage and fat cover after finishing in the feedlot for 180 days [29]. Carcass subcutaneous fat cover is essential to reduce the risk of cold shortening that creates myofibrillar toughening resulting in decreased meat tenderness [42]. Thus, cattle are feedlot finished on energy-dense diets to improve the subcutaneous fat depth [28,29]. Backfat thickness of concentrate finished cattle is reported to be higher than in their forage finished counterparts in some studies [28,34,53], but not others [74]. This can be explained by the increased energy intake for fat synthesis when cattle are fed energy-dense diets during finishing compared to the backgrounding pasture diets [19]. In addition, the extensive fermentation of forage diets in the rumen promotes acetate production as the primary source of carbon that reduces lipogenesis, while concentrates increase glucose flow into the duodenum, thereby promoting lipogenesis [28]. 

In this study, while there were no differences between diets in LW and carcass fatness, significant differences between feedlot finished and unfinished steers were recorded. This tallies with the expected trends in dressing percentage between finished and unfinished steers and between diets. Blanco et al. [74] reported that supplementing steers with barley improved dressing percentage and HCW compared to steers fed on lucerne hay. Finishing Mertolenga steers for 100 days increased backfat thickness from 1.4 to 4.2 mm [75], but bulls finished to similar weights had similar HCW and dressing percentages [28]. Dressing percentage is influenced by breed, liveweight, carcass fatness and time off water [76,77]. Our findings indicate that backgrounding tropical crossbred beef cattle on desmanthus alone or mixed with other high-quality legumes may result in healthy meat, and palatability can be improved by feedlot finishing for a short period. These findings are essential for beef cattle producers in the environmentally harsh tropical and subtropical northern Australian regions where desmanthus is one of the few legume forages that have adapted, established and persisted over several years.

## 4. Materials and Methods

The backgrounding phase of this study took place at the CSIRO Lansdown Research Station, Queensland, Australia, between March and July 2020, while the finishing phase was carried out at a commercial feedlot 17 km from the research station from August to October 2020. The mean monthly minimum and maximum temperatures and rainfall were 15.5 °C, 24.9 °C and 26.0 mm, respectively, while average minimum and maximum relative humidity were 55 and 65% during the experimental period. All procedures in this study were carried out according to the CSIRO Animal Ethics Committee approved guidelines (approval number 2019-38) and the Australian code of practice for the care and use of animals for scientific purposes [78].

### 4.1. Animals, Diets and Experimental Design

Sample size determination, animal management and treatments are described in detail in our previous study (under review). In brief, 48 Brahman, Charbray and Droughtmaster crossbred steers were backgrounded on Rhodes grass hay supplemented with incremental proportions of freshly cut desmanthus for 140 days in a completely randomised design. Desmanthus (comprised of three species, namely *D. virgatus* cv. JCU2, *D. bicornutus* cv. JCU4 and *D. leptophyllus* cv. JCU7 (Agrimix Pastures Pty Ltd., Ferny Hills DC, QLD, Australia) in equal proportions) accounted for 0, 15%, 30% or 45% DM and varying proportions of lucerne hay were added to the 0, 15%, 30% desmanthus diets to ensure that the diets were isonitrogenous to the 45% desmanthus diet (Table 4 and Table 5). Steers aged 28–33 months old weighed 332 ± 21 kg and 429 ± 31 kg at the start and end of the backgrounding phase, respectively, and they were group-housed in 12 outdoor pens with four steers in each pen and three pens per treatment. Each pen measured 60 m^2^ and was fitted with 18 m^2^ shade, water trough and 4 m by 1 m feed trough. The pen boundaries were portable metallic panels, and the floor was made of roadbase grade stone topped with crusher dust compacted and covered with soil. At the end of the backgrounding phase steers were separated into two groups based on liveweight. The two heaviest (453 ± 15 kg) steers per pen were slaughtered without finishing, whereas the other two steers (406 ± 25 kg) were transferred and fed at a commercial feedlot in accordance with the standard feedlot finishing rations. During the finishing phase, steers were housed in one outdoor pen allowing 11 m^2^/head stocking density and were allowed unlimited access to clean water and feed. The feedlot finishing phase lasted for 95 days following the 70–100 days finishing phase commonly practiced in Australia [79]. After finishing, steers were transported to a nearby commercial abattoir for slaughter and graded according to AUS-MEAT standards [80].

### 4.2. Loin Eye Muscle Biopsy and Carcass Sampling

To determine the IMF, FMP and FA composition, biopsy samples were collected from the loin eye muscle at the start and end of the backgrounding phase from the 12th–13th rib interface based on the procedure described by Malau-Aduli et al. [82]. In summary, steers were restrained in a crush and the hair at and around the 12th–13th rib interface was clipped. The clipped area was prepared aseptically and desensitised with a local anesthetic (Ilium Lignocaine 20^®^, Troy Animal Healthcare, Glendenning, New South Wales, Australia) and 3 g of muscle was taken. The incision was closed using absorbable monofilament suture material. Steers were prophylactically treated with Depocillin^®^ (MSD Animal Health, Macquarie Park, New South Wales, Australia) both rounds and Metacam^®^ 20 mg/mL Solution for Injection (Boehringer Ingelheim Animal Health, Australia Pty Ltd., North Ryde, New South Wales, Australia) the second round of biopsies. Cetrigen^®^ (Virbac, Australia Pty Ltd., Milperra, New South Wales, Australia) antibacterial wound aerosol and insect repellent was sprayed on and directly around the wound to prevent secondary infection and keep flies at bay. Steers were taken back to their respective pens and monitored twice daily until the wounds healed and no post-operative complications were recorded. Biopsy samples for the baseline analysis were taken from the left side of the animal and on the right side at the end of the backgrounding phase. Samples were placed on dry ice immediately after collection, transported to the laboratory and stored at −20 °C until analysis. For the carcasses, 10 g of the loin eye muscle were collected at the 12th and 13th ribs interface of the chilled carcasses 12 h after slaughter and stored at −20 °C until analysis.

### 4.3. IMF, FMP and FA Composition Analysis

The IMF content of biopsy and carcass samples was extracted and purified according to the modified method of Folch et al. [83] as described by Flakemore et al. [84]. The procedure involved muscle sample homogenisation, overnight extraction using CHCl_3_: MeOH (2:1 *v*/*v*) solvent mixture, phase separation with 5 mL of 10% KCl, manual removal of the upper inorganic layer and heat evaporation using porcelain crucibles to obtain the fat content. The IMF percentage was calculated as:

(Crucible with fat weight (g)—empty crucible weight (g))/sample weight (g) × 100(1)

The FMP was analysed using the slip melting point method [85] as described by Pewan et al. [86]. Briefly, fat extracted for IMF content determination was melted in an oven at 100 °C for 1–2 min. The melted fat was transferred into capillary tubes and placed in a refrigerator at 4 °C for 10 min to allow the fat to solidify. Fat level was marked with a permanent pen and the capillary tube attached to the thermometer and suspended in a glass beaker with 80 mL deionised H_2_O placed on a heating block. The heating block was gradually heated and the fat level closely monitored until fat melted and ‘slipped’ above the mark. The ‘slip point’ temperature was recorded as the FMP. 

The FA composition of the loin eye muscle samples was analysed using the gas chromatography–mass spectrometry procedure previously reported by Malau-Aduli et al. [87]. In summary, FA analysis procedure included three steps. The first step was the lipid extraction: Total lipids of wet unground 1 g muscle samples were extracted according to a modified Bligh and Dyer protocol [88]. The protocol entailed a single-phase overnight extraction with CHCl3:MeOH:H_2_O at 1:2:0.8 *v*/*v*, phase separation by addition of CHCl3:saline Milli-Q H_2_O at 1:1 *v*/*v* and rotary evaporation of the chloroform phase at 40 °C to obtain total lipids. The second step was methylation: Total lipids aliquots were transmethylated in MeOH:CHCl_3_:HCl at 10:1:1 *v*/*v* for 2 h at 80 °C. Milli-Q H_2_O (1 mL) was added, FA methyl esters extracted with hexane:chloroform at 4:1 *v*/*v* and flushed with nitrogen gas. The final step was the FA quantification: The extracted FA methyl esters were topped up to 1500 µL volume with an internal injection reference standard (19:0). The FA methyl esters analysis was carried out using a 7890B gas chromatograph (GC) (Agilent Technologies, Palo Alto, CA, USA) equipped with an Agilent Technologies 7683 B Series autosampler, a split/splitless injector, EquityTM-1 fused 15 m silica capillary column with 0.1 mm internal diameter and 0.1-µm film thickness (Supelco, Bellefonte, PA, USA) and a flame ionisation detector. The carrier gas was helium and initial oven temperature of 120 °C that was increased to 270 °C at 10 °C/min rate and then to 310 °C at 5 °C/min. FA peaks were quantified using the Agilent Technologies ChemStation software (Palo Alto, CA, USA). The FA identities were confirmed using a GC-mass spectrometric analysis with a Thermo Scientific 1310 GC attached to a TSQ triple quadropole (Thermo Fisher Scientific, Milan, Italy) PTV injector and Thermo Scientific Xcalibur^TM^ software (Austin, TX, USA). The GC working conditions were as previously reported by Miller et al. [89]. FA percentages (%FA) and FA contents (FA mg/100 g muscle) were calculated as [90]:

%FA = (individual FA area) × (100)/(sum total area of FA)(2)

FA (mg/100 g) = Total lipid (g/100 g) × 0.916 × (%FA)/100) × 1000(3)

where 0.916 was the lipid conversion factor based on the assumption that beef lipid contain ≈12% phospholipids and ≈1% cholesterol [91].

### 4.4. Statistical Analysis

Data were analysed using the Statistical Analysis System software version 9.4 (SAS Institute, Cary, NC, USA). Initial data screening was carried out by computing summary statistics of means, standard deviations, minimum and maximum values to examine data for entry errors and outliers. Data were analysed by linear mixed model (PROC MIXED) procedure with the fixed effect of backgrounding diet (0, 15%, 30% and 45% desmanthus diets) and pen nested within diet as a random effect to determine the effect of backgrounding diet on IMF, FMP, FA composition and feedlot growth performance. The same model was used to examine the effect of backgrounding diet, feedlot finishing (feedlot finished vs. unfinished) and their interactions on carcass characteristics and FA composition. Baseline measurements of IMF, FMP, FA composition were included as covariates in the model. When the effect of diet was significant (*p* < 0.05), orthogonal polynomial contrasts were performed to test for linear, quadratic and cubic responses to increasing desmanthus proportions. Significant interactions of backgrounding diet and feedlot finishing were separated using the Tukey-Kramer pairwise comparison test. The quadratic and cubic responses were eventually dropped from the model because they were not significant.

## 5. Conclusions

Backgrounding steers on grass forage augmented with incremental proportions of desmanthus resulted in similar muscle IMF (intramuscular fat), FMP (fat melting point) and FA (fatty acids) composition. Growth performance during finishing and ultimate carcass quality were comparably similar in all steers. Hence, our hypothesis that steers backgrounded on isonitrogenous diets augmented with incremental proportions of desmanthus will produce similar carcass characteristics and FA composition was accepted. Feedlot finishing increased carcass weight and fatness and maintained the n-6/n-3 ratio below 4.0. These findings indicate that backgrounding tropical beef cattle on desmanthus forage and finishing them in the feedlot for a short period (95 days) results in healthy and highly palatable meat. Further studies are required to examine the effect of backgrounding tropical beef cattle with incremental proportions of desmanthus forage on the expression of lipogenic genes associated with fat metabolism and meat eating quality.

## Figures and Tables

**Table 1:** Effect of desmanthus supplementation on intramuscular fat, fat melting point and fatty acids composition (least square mean) of the loin eye muscle of tropical crossbred beef cattle.

Variable ^1^	Desmanthus Proportion in the Diet (%)				SEM ^2^	*p*-Value
	0	15	30	45		
IMF (%)	2.6	2.3	2.1	2.3	0.11	0.50
FMP (°C)	43.1	43.5	43.1	44.5	0.71	0.94
Fatty Acids (mg/100g)						
C13:0	0.2	0.1	0.3	0.1	0.03	0.51
C14:0	9.7	12.8	14.3	15.6	2.11	0.77
C14:1	1.4	2.1	2.1	2.8	0.40	0.69
C15:0	3.5	4.9	4.0	6.0	0.64	0.54
C16:0	156.9	209.9	203.5	215.7	21.83	0.67
C16:1	23.8	28.2	33.5	30.9	3.16	0.70
C17:0	9.4	11.0	12.4	11.7	1.06	0.78
C17:1	9.2	10.3	11.3	10.7	0.96	0.88
C18:0	102.7	135.2	137.1	139.6	11.40	0.60
C18:1 (Oleic)	190.0	250.3	288.6	258.8	28.48	0.58
CLA	3.1	4.1	5.4	4.9	0.49	0.48
C18:2n-6 (LA)	44.7	52.8	48.1	54.8	1.47	0.25
C18:3n-3 (ALA)	15.3	17.1	15.8	16.9	0.45	0.47
C18:3n-6	0.4	0.6	0.6	0.6	0.04	0.35
C18:4n-3	2.4	2.6	1.9	2.3	0.11	0.25
C19:1	1.4	1.6	2.1	1.9	0.21	0.64
C20:0	0.8	1.2	1.3	1.3	0.13	0.48
C20:1	1.6	2.2	2.3	2.5	0.22	0.38
C20:2n-6	0.7	1.1	0.9	0.9	0.06	0.30
C20:3	9.9	10.8	10.2	9.6	0.26	0.45
C20:4n-3	3.1	3.1	2.9	2.9	0.10	0.78
C20:4n-6 (ARA)	26.3	26.7	26.6	25.0	0.85	0.90
C20:5n-3 (EPA)	9.7	9.9	9.5	8.6	0.31	0.43
C21:0	0.2	0.3	0.3	0.3	0.01	0.98
C21:5n-3	0.2	0.3	0.3	0.2	0.02	0.56
C22:0	1.4	1.5	2.0	1.6	0.06	0.04 ^3^
C22:1	0.8	0.8	0.9	0.7	0.04	0.74
C22:4n-6	1.8	2.0	2.0	1.9	0.05	0.58
C22:5n-6	0.5	0.5	0.4	0.5	0.01	0.72
C22:5n-3 (DPA)	13.9	15.0	14.0	13.4	0.48	0.76
C22:6n-3 (DHA)	2.8	2.3	2.2	2.1	0.11	0.20
C23:0	1.6	1.6	1.8	1.6	0.04	0.60
C24:0	2.4	2.3	3.0	2.7	0.17	0.38
C24:1	1.1	1.0	1.2	1.1	0.04	0.60
EPA + DHA	12.5	12.3	11.8	10.7	0.40	0.41
EPA + DPA + DHA	26.4	27.2	25.8	24.1	0.83	0.62
Total FA	644.5	816.5	859.3	852.5	71.66	0.59
∑SFA	285.7	367.8	381.8	392.6	37.07	0.65
∑MUFA	229.7	296.1	342.1	310.7	33.35	0.60
∑PUFA	134.3	150.6	141.8	144.6	3.82	0.59
∑n-3 PUFA	47.4	50.3	49.2	46.2	1.28	0.70
∑n-6 PUFA	74.3	83.9	76.7	83.8	2.33	0.58
PUFA/SFA	0.6	0.5	0.4	0.5	0.03	0.59
n-6/n-3 PUFA	1.5	1.6	1.7	1.8	0.02	0.01 ^3^

^1^ IMF, intramuscular fat; FMP, fat melting point; CLA, conjugated linoleic acid; LA, linoleic acid; ALA, α-linolenic acid; ARA, arachidonic acid; EPA, eicosapentaenoic acid; DPA, docosapentaenoic acid; DHA, docosahexaenoic acid; Total FA, sum of all fatty acids measured; ΣSFA, total saturated fatty acids; ΣMUFA, total monounsaturated fatty acids; ΣPUFA, total polyunsaturated fatty acids. ΣSFA = 14:0 + 15:0 + 16:0 +17:0 +18:0 + 20:0 + 21:0; ΣMUFA = 14:1 + 16:1n-13t + 16:1n-9 + 16:1n-7 + 16:1n-7t + 16:1n-5c + 17:1n-8+a17:0 + 18:1n-7t + 18:1n-5 + 18:1n-7 + 18:1n-9 + 18:1a, + 18:1b + 18:1c + 19:1a + 19:1b + 20:1n-11 + 20:1n-9 + 20:1n-7 + 20:1n-5 + 22:1n-9 + 22:1n-11 + 24:1n-9; ΣPUFA = 18:4n-3 + 18:3n-6 + 18:2n-6 + 18:3n-3 + 20:2n-6 + 20:3 + 20:3n-6 + 20:4n-3 + 20:4n-6 +20:5n-3 + 22:4n-6 + 22:5n-3 + 22:5n-6 + 22:6n-3; Σn-3 PUFA = 18:3n-3 + 18:4n-3 + 20:4n-3 + 20:5n-3 + 22:5n-3 + 22:6n-3; Σn-6 PUFA = 18:2n-6 + 18:3n-6 + 20:2n-6 + 20:3n-6 + 20:4n-6 + 22:4n-6 + 22:5n-6. ^2^ SEM, standard error of the mean. ^3^ Linear *p*-Value.

**Table 2:** Carcass intramuscular fat content, fat melting point and fatty acid profiles of feedlot-finished and unfinished steers.

Variable ^1^	Unfinished				Feedlot Finished					*p*-Value ^2^		
	0	15	30	45	0	15	30	45	SEM	D	F	D*F
IMF (%)	3.5	2.6	2.4	2.2	4.5	4.5	4.5	4.5	0.18	0.33	0.01	0.29
FMP (°C)	44.3	42.0	43.4	45.1	41.6	43.6	39.0	40.6	0.48	0.49	0.01	0.04
Fatty Acids (mg/100g)												
C13:0	0.3	0.2	0.5	0.3	0.0	0.0	0.0	0.0	0.04	0.27	0.01	0.27
C14:0	3.9	18.9	8.9	18.5	16.6	13.4	36.3	17.5	2.63	0.42	0.07	0.06
C14:1	0.3	3.5	1.3	3.0	3.0	2.8	3.6	3.3	0.55	0.74	0.03	0.04
C15:0	2.2	5.2	4.3	3.9	3.2	3.6	7.7	3.8	0.55	0.30	0.46	0.25
C16:0	119.8	280.6	177.5	262.9	373.9	236.5	413.1	271.3	26.22	0.92	0.01	0.03
C16:1	20.5	38.8	27.6	39.2	58.1	30.7	72.9	36.9	4.34	0.62	0.02	0.04
C17:0	7.2	13.2	11.3	14.2	12.2	10.4	21.5	12.2	1.17	0.32	0.20	0.09
C17:1	7.2	11.9	9.9	12.7	20.4	11.4	23.4	13.9	1.35	0.59	0.01	0.09
C18:0	86.8	167.7	140.4	165.6	169.1	155.1	214.9	170.5	11.38	0.54	0.06	0.22
C18:1 (Oleic)	151.4	331.0	244.5	316.9	581.6	356.4	649.0	407.1	39.88	0.77	0.01	0.08
CLA	2.5	4.4	5.5	5.5	6.4	5.5	8.9	6.4	0.60	0.35	0.04	0.68
C18:2n-6 (LA)	43.0	48.6	53.1	48.9	85.1	74.2	81.6	80.8	2.73	0.55	0.01	0.22
C18:3n-3 (ALA)	14.8	15.1	16.5	15.4	6.8	7.4	6.5	7.4	0.73	0.93	0.01	0.76
C18:3n-6	0.2	0.7	0.4	0.6	1.0	0.9	1.1	0.9	0.06	0.64	0.01	0.06
C18:4n-3	2.3	2.9	2.7	2.3	3.4	2.7	3.0	2.8	0.10	0.68	0.07	0.12
C19:1	1.0	2.0	1.8	2.4	2.3	1.3	2.8	1.6	0.22	0.61	0.64	0.15
C20:0	0.7	1.4	1.2	1.7	1.8	1.3	1.6	1.2	0.14	0.93	0.38	0.20
C20:1	1.2	2.4	2.3	3.4	4.3	3.2	3.9	3.5	0.30	0.87	0.01	0.19
C20:2n-6	0.6	1.2	1.0	1.1	1.5	1.0	1.4	1.1	0.08	0.94	0.08	0.05
C20:3	10.2	10.5	11.2	9.2	12.5	9.8	12.4	13.8	0.32	0.14	0.01	0.01
C20:4n-3	3.4	2.9	3.1	2.7	1.8	1.9	1.6	2.0	0.12	0.73	0.01	0.13
C20:4n-6 (ARA)	25.8	22.5	27.8	21.3	31.1	29.5	28.8	32.6	0.76	0.44	0.01	0.03
C20:5n-3 (EPA)	9.2	8.7	9.6	7.4	8.8	8.0	7.1	8.3	0.25	0.47	0.16	0.11
C21:0	0.2	0.3	0.3	0.3	0.1	0.1	0.2	0.1	0.02	0.93	0.01	0.48
C21:5n-3	0.3	0.3	0.3	0.2	0.4	0.3	0.3	0.3	0.01	0.17	0.06	0.01
C22:0	1.3	1.5	1.6	1.5	1.9	1.5	1.6	1.9	0.05	0.55	0.01	0.02
C22:1	0.7	0.8	0.9	0.7	0.9	0.9	0.7	0.7	0.04	0.59	0.90	0.11
C22:4n-6	1.8	2.0	2.2	2.0	3.6	3.5	3.4	3.7	0.01	0.89	0.01	0.37
C22:5n-6	0.5	0.5	0.5	0.4	0.7	0.6	0.5	0.6	0.13	0.50	0.01	0.17
C22:5n-3 (DPA)	13.6	13.7	15.4	12.8	16.6	15.9	14.2	14.9	0.37	0.60	0.04	0.20
C22:6n-3 (DHA)	2.5	2.0	2.2	1.8	2.7	2.1	2.2	2.0	0.09	0.01 ^3^	0.38	0.97
C23:0	1.6	1.7	2.0	1.8	1.4	1.3	1.2	1.4	0.05	0.81	0.01	0.06
C24:0	1.6	1.7	1.9	1.8	2.0	1.6	1.7	1.8	0.05	0.68	0.89	0.24
C24:1	1.0	1.1	1.2	1.2	1.1	0.9	0.9	0.9	0.04	0.92	0.07	0.41
EPA+DHA	11.7	10.7	11.8	9.1	11.3	10.1	9.5	10.3	0.30	0.19	0.34	0.18
EPA+DPA+DHA	25.2	24.4	27.2	21.9	27.9	26.0	23.6	25.1	0.61	0.37	0.41	0.18
Total FA	523.7	1005.1	787.5	997.7	1513.8	1006.5	1635.5	1128.8	93.38	0.84	0.01	0.07
∑SFA	223.9	472.2	349.0	473.4	668.5	422.6	707.0	479.9	44.25	0.90	0.02	0.06
∑MUFA	184.7	391.5	289.6	381.7	675.7	407.2	766.0	468.1	46.70	0.75	0.01	0.07
∑PUFA	129.7	139.8	151.7	132.5	186.7	164.3	175.3	177.8	4.20	0.67	0.01	0.19
∑n-3 PUFA	45.9	45.7	49.9	42.7	40.3	38.2	34.9	37.7	1.14	0.77	0.01	0.27
∑n-6 PUFA	72.0	75.7	85.1	73.4	123.0	109.6	116.8	119.7	3.49	0.52	0.01	0.23
PUFA/SFA	0.6	0.4	0.5	0.4	0.4	0.4	0.3	0.4	0.03	0.59	0.04	0.06
n-6/n-3 PUFA	1.5	1.7	1.7	1.8	3.1	2.9	3.5	3.1	0.12	0.26	0.01	0.28

^1^ Abbreviations are as depicted in Table 1. ^2^ D, diet; F, finishing, D*F, diet and finishing interactions. ^3^ Linear *p*-Value.

**Table 3:** Effect of desmanthus proportion and feedlot finishing on slaughter weight and carcass characteristics.

Variable ^1^	Unfinished				Finished					*p*-Value ^2^		
	0	15	30	45	0	15	30	45	SEM	D	F	D*F
Slaughter weight (kg)	463.8	457.7	443.5	447.5	532.8	550.5	558.0	557.3	8.11	0.96	0.01	0.21
HCW (kg)	225.3	219.0	216.7	220.8	267.2	275.0	274.2	274.9	4.42	0.98	0.01	0.63
Dressing percentage (%)	52.8	52.0	53.1	53.6	54.5	54.3	53.4	53.6	0.26	0.86	0.04	0.30
P8 fat (mm)	4.3	4.8	3.3	3.0	9.7	9.2	7.5	8.7	0.50	0.28	0.01	0.84

^1^ HCW, hot carcass weight; P8 fat, Subcutaneous fat depth at the P8 (rump) site. ^2^ D, diet; F, finishing, D*F, diet and finishing interaction.

**Table 4:** Diet chemical composition, DMI and steers growth performance during backgrounding.

Variable ^1^	Desmanthus Proportion in the Diet (%)			
	0	15	30	45
DM (%)	87.3	68.4	56.9	48.6
CP	11.6	11.6	11.5	11.4
NDF	65.2	64.9	64.6	64.4
ADF	40.1	40.4	40.8	41.1
Hemicellulose	25.1	24.4	23.8	23.3
ME (MJ/kg DM) ^2^	8.1	7.9	7.6	7.4
DMI (kg/head)	8.8	8.5	8.2	7.6
Initial LW (kg)	332.0	330.2	332.7	332.8
Final LW (kg)	434.1	437.8	427.5	420.1
ADG (kg/day)	0.63	0.66	0.57	0.53

^1^ CP, crude protein; NDF, neutral detergent fibre; ADF, acid detergent fibre; ME, metabolisable energy; DMI. Dry matter intake; ADG, average daily gain; LW, liveweight. Data are presented in percentage of dry matter (DM) unless otherwise stated. ^2^ Estimated from in vitro dry matter digestibility as DMD × 0.172 − 1.707 [81].

**Table 5:** Fatty acids composition (% of total fatty acids) of the Rhodes grass, lucerne and desmanthus forages.

Variable ^1^		Forage ^2^				
	Rhodes Grass	Lucerne	JCU2	JCU4	JCU7	Desmanthus
C14:0	0.6	0.6	0.3	0.3	0.3	0.3
C15:0	0.9	1.6	0.4	0.3	0.5	0.4
C16:0	31.3	26.4	20.5	20.2	20.1	20.3
C16:1	2.8	3.9	3.2	2.7	2.7	2.9
C17:0	2.5	1.7	1.1	1.1	1.2	1.1
C17:1	0.3	0.2	0.2	0.1	0.2	0.2
C18:0	4.0	5.5	5.1	5.1	4.9	5.0
C18:1	3.3	4.0	5.3	5.4	6.4	5.7
C18:2n-6 (LA)	14.5	16.4	18.8	20.8	21.5	20.4
C18:3n-3 (ALA)	25.6	26.5	36.9	34.4	33.4	34.9
C18:3n-6	1.9	1.8	0.9	0.7	0.8	0.8
CLA	0.3	0.6	0.1	0.4	0.2	0.2
C19:1	0.0	0.2	0.4	0.2	0.1	0.2
C20:0	1.5	1.4	1.1	1.1	0.8	1.0
C20:1	0.6	0.7	0.4	0.6	0.4	0.5
C20:2n-6	0.0	0.1	0.1	0.1	0.1	0.1
C20:3	0.6	0.6	0.2	0.2	0.3	0.2
C20:4n-3	0.5	0.5	0.2	0.2	0.2	0.2
C20:5n-3 (EPA)	0.1	0.1	0.0	0.0	0.0	0.0
C21:0	0.4	0.5	0.4	0.4	0.4	0.4
C21:5n-3	0.2	0.3	0.1	0.1	0.1	0.1
C22:0	2.4	1.8	1.5	1.8	1.9	1.7
C22:1	0.3	0.9	0.1	0.4	0.2	0.2
C22:4n-6	0.0	0.1	0.1	0.2	0.1	0.1
C22:5n-3 (DPA)	0.9	0.2	0.0	0.0	0.0	0.0
C22:5n-6	0.0	0.2	0.0	0.1	0.0	0.0
C22:6n-3 (DHA)	0.5	0.3	0.2	0.3	0.1	0.2
C23:0	0.7	0.7	0.4	0.4	0.6	0.5
C24:0	2.6	1.8	1.9	2.0	2.6	2.2
C24:1	0.3	0.2	0.2	0.1	0.1	0.1
EPA+DHA	0.6	0.4	0.2	0.3	0.1	0.2
EPA+DPA+DHA	1.5	0.6	0.2	0.3	0.1	0.2
∑SFA	47.1	42.1	32.7	32.8	33.2	32.9
∑MUFA	7.7	10.1	9.7	9.6	10.1	9.8
∑PUFA	45.2	47.8	57.6	57.6	56.7	57.3
∑n-3 PUFA	27.9	27.9	37.4	35.1	33.8	35.4
∑n-6 PUFA	16.4	18.7	19.9	21.9	22.5	21.4
PUFA/SFA	1.0	1.1	1.8	1.8	1.7	1.8
n-6/n-3 PUFA	0.6	0.7	0.5	0.6	0.7	0.6

^1^ Abbreviations are as defined in Table 1. ^2^ JCU2, *D. virgatus*; JCU4, *D. bicornutus*; JCU7, *D. leptophyllus*; desmanthus, average of the three desmanthus cultivars.

## Data Availability

The data presented in this study are available from the corresponding author on request.

## References

[1] Organisation for Economic Co-Operation and Development (OECD) (2021). Meat Consumption (indicator). https://data.oecd.org/agroutput/meat-consumption.htm.

[2] FAO (2021). Meat Market Review: Overview of Global Meat Market Developments in 2020.

[3] Cabrera M.C., Saadoun A. (2014). An overview of the nutritional value of beef and lamb meat from South America. Meat Sci..

[4] Troy D.J., Tiwari B.K., Joo S. (2016). Health implications of beef intramuscular fat consumption. Korean J. Food Sci. Anim. Resour..

[5] Arnett D.K., Blumenthal R.S., Albert M.A., Buroker A.B., Goldberger Z.D., Hahn E.J., Himmelfarb C.D., Khera A., Lloyd-Jones D., McEvoy J.W. (2019). 2019 ACC/AHA Guideline on the Primary Prevention of Cardiovascular Disease: A Report of the American College of Cardiology/American Heart Association Task Force on Clinical Practice Guidelines. Circulation.

[6] Bouvard V., Loomis D., Guyton K.Z., Grosse Y., El Ghissassi F., Benbrahim-Tallaa L., Guha N., Mattock H., Straif K., Stewart B.W. (2015). Carcinogenicity of consumption of red and processed meat. Lancet Oncol..

[7] Johnston B.C., Zeraatkar D., Han M.A., Vernooij R.W.M., Valli C., El Dib R., Marshall C., Stover P.J., Fairweather-Taitt S., Wójcik G. (2019). Unprocessed red meat and processed meat consumption: Dietary guideline recommendations from the Nutritional Recommendations (NutriRECS) Consortium. Ann. Intern. Med..

[8] Woollett L.A., Spady D.K., Dietschy J.M. (1992). Saturated and unsaturated fatty acids independently regulate low density lipoprotein receptor activity and production rate. J. Lipid Res..

[9] Krauss R.M., Kris-Etherton P.M. (2020). Public health guidelines should recommend reducing saturated fat consumption as much as possible: Debate consensus. Am. J. Clin. Nutr..

[10] Van Vliet S., Provenza F.D., Kronberg S.L. (2021). Health-promoting phytonutrients are higher in grass-fed meat and milk. Front. Sustain. Food Syst..

[11] Schulze M.B., Minihane A.M., Saleh R.N.M., Risérus U. (2020). Intake and metabolism of omega-3 and omega-6 polyunsaturated fatty acids: Nutritional implications for cardiometabolic diseases. Lancet Diabetes Endocrinol..

[12] Simopoulos A.P. (2016). An increase in the Omega-6/Omega-3 fatty acid ratio increases the risk for obesity. Nutrients.

[13] Wood J.D., Richardson R.I., Nute G.R., Fisher A.V., Campo M.M., Kasapidou E., Sheard P.R., Enser M. (2003). Effects of fatty acids on meat quality: A review. Meat Sci..

[14] Hwang Y.H., Bakhsh A., Ismail I., Lee J.G., Joo S.T. (2018). Effects of intensive alfalfa feeding on meat quality and fatty acid profile of Korean native black goats. Korean J. Food Sci. Anim. Resour..

[15] Flakemore A.R., Malau-Aduli B.S., Nichols P.D., Malau-Aduli A.E.O. (2017). Degummed crude canola oil, sire breed and gender effects on intramuscular long-chain omega-3 fatty acid properties of raw and cooked lamb meat. J. Anim. Sci. Technol..

[16] Van Le H., Nguyen D.V., Vu Nguyen Q., Malau-Aduli B.S., Nichols P.D., Malau-Aduli A.E.O. (2019). Fatty acid profiles of muscle, liver, heart and kidney of Australian prime lambs fed different polyunsaturated fatty acids enriched pellets in a feedlot system. Sci. Rep..

[17] Girard M., Dohme-Meier F., Silacci P., Ampuero Kragten S., Kreuzer M., Bee G. (2016). Forage legumes rich in condensed tannins may increase n-3 fatty acid levels and sensory quality of lamb meat. J. Sci. Food Agric..

[18] Neves D.S.B., Rodrigues Silva R., da Silva F.F., Santos L.V., Filho G.A., de Souza S.O., da Santos M.C., Rocha W.J., da Silva A.P.G., de Melo Lisboa M. (2018). Increasing levels of supplementation for crossbred steers on pasture during the dry period of the year. Trop. Anim. Health Prod..

[19] Scollan N., Hocquette J.F., Nuernberg K., Dannenberger D., Richardson I., Moloney A. (2006). Innovations in beef production systems that enhance the nutritional and health value of beef lipids and their relationship with meat quality. Meat Sci..

[20] Scollan N.D., Dannenberger D., Nuernberg K., Richardson I., MacKintosh S., Hocquette J.F., Moloney A.P. (2014). Enhancing the nutritional and health value of beef lipids and their relationship with meat quality. Meat Sci..

[21] Kronberg S.L., Scholljegerdes E.J., Barceló-Coblijn G., Murphy E.J. (2007). Flaxseed treatments to reduce biohydrogenation of α-linolenic acid by rumen microbes in cattle. Lipids.

[22] Alves S.P., Francisco A., Costa M., Santos-Silva J., Bessa R.J.B. (2017). Biohydrogenation patterns in digestive contents and plasma of lambs fed increasing levels of a tanniferous bush (*Cistus ladanifer* L.) and vegetable oils. Anim. Feed Sci. Technol..

[23] Liu C., Xu C., Qu Y., Guo P., Ma Y., Wang B., Zhang H., Luo H. (2021). Effect of alfalfa (*Medicago sativa* L.) saponins on meat color and myoglobin reduction status in the longissimus thoracis muscle of growing lambs. Anim. Sci. J..

[24] Schlink A.C., Burt R.L. (1993). Assessment of the chemical composition of selected tropical legume seeds as animal feed. Trop. Agric..

[25] Suybeng B., Charmley E., Gardiner C.P., Malau-Aduli B.S., Malau-Aduli A.E.O. (2019). Methane emissions and the use of desmanthus in beef cattle production in Northern Australia. Animals.

[26] Suybeng B., Charmley E., Gardiner C.P., Malau-Aduli B.S., Malau-Aduli A.E.O. (2020). Supplementing Northern Australian beef cattle with desmanthus tropical legume reduces in-vivo methane emissions. Animals.

[27] Department of Agriculture and Fisheries (DAF) (2018). The Queensland Beef Supply Chain.

[28] Blanco M., Casasús I., Ripoll G., Panea B., Albertí P., Joy M. (2010). Lucerne grazing compared with concentrate-feeding slightly modifies carcase and meat quality of young bulls. Meat Sci..

[29] Kurve V.P., Joseph P., Williams J.B., Kim T.J., Boland H., Smith T., Schilling M.W. (2016). The effect of feeding native warm season grasses in the stocker phase on the carcass quality, meat quality, and sensory attributes of beef loin steaks from grain-finished steers. Meat Sci..

[30] Woods V.B., Fearon A.M. (2009). Dietary sources of unsaturated fatty acids for animals and their transfer into meat, milk and eggs: A review. Livest. Sci..

[31] Yang A., Larsen T.W., Smith S.B., Tume R.K. (1999). Δ9 Desaturase activity in bovine subcutaneous adipose tissue of different fatty acid composition. Lipids.

[32] Dierking R.M., Kallenbach R.L., Grün I.U. (2010). Effect of forage species on fatty acid content and performance of pasture-finished steers. Meat Sci..

[33] Kennedy P.C., Dawson L.E.R., Lively F.O., Steen R.W.J., Fearon A.M., Moss B.W., Kilpatrick D.J. (2018). Effects of offering lupins/triticale and vetch/barley silages alone or in combination with grass silage on animal performance, meat quality and the fatty acid composition of lean meat from beef cattle. J. Agric. Sci..

[34] Duckett S.K., Neel J.P.S., Lewis R.M., Fontenot J.P., Clapham W.M. (2013). Effects of forage species or concentrate finishing on animal performance, carcass and meat quality. J. Anim. Sci..

[35] Chail A., Legako J.F., Pitcher L.R., Griggs T.C., Ward R.E., Martini S., MacAdam J.W. (2016). Legume finishing provides beef with positive human dietary fatty acid ratios and consumer preference comparable with grain-finished beef. J. Anim. Sci..

[36] Freitas A.K.d., Lobato J.F.P., Cardoso L.L., Tarouco J.U., Vieira R.M., Dillenburg D.R., Castro I. (2014). Nutritional composition of the meat of Hereford and Braford steers finished on pastures or in a feedlot in southern Brazil. Meat Sci..

[37] Hwang Y.H., Joo S.T. (2017). Fatty acid profiles, meat quality, and sensory palatability of grain-fed and grass-fed beef from Hanwoo, American, and Australian crossbred cattle. Korean J. Food Sci. Anim. Resour..

[38] Wood J.D., Enser M., Fisher A.V., Nute G.R., Richardson R.I., Sheard P.R. (1999). Manipulating meat quality and composition. Proc. Nutr. Soc..

[39] Smith S.B. (2016). Marbling and its nutritional impact on risk factors for cardiovascular disease. Korean J. Food Sci. Anim. Resour..

[40] Malau-Aduli A.E.O., Edriss M.A., Siebert B.D., Bottema C.D.K., Pitchford W.S. (2000). Breed differences and genetic parameters for melting point, marbling score and fatty acid composition of lot-fed cattle. J. Anim. Physiol. Anim. Nutr..

[41] Pitchford W.S., Deland M.P.B., Siebert B.D., Malau-Aduli A.E.O., Bottema C.D.K. (2002). Genetic variation in fatness and fatty acid composition of crossbred cattle. J. Anim. Sci..

[42] Turk S.N., Smith S.B. (2009). Carcass fatty acid mapping. Meat Sci..

[43] May S.G., Sturdivant C.A., Lunt D.K., Miller R.K., Smith S.B. (1993). Comparison of sensory characteristics and fatty acid composition between Wagyu crossbred and Angus steers. Meat Sci..

[44] Perry D., Nicholls P.J., Thompson J.M. (1998). The effect of sirebreed on the melting point and fatty acid composition of subcutaneous fat in steers. J. Anim. Sci..

[45] Alfaia C.P.M., Alves S.P., Martins S.I.V., Costa A.S.H., Fontes C.M.G.A., Lemos J.P.C., Bessa R.J.B., Prates J.A.M. (2009). Effect of the feeding system on intramuscular fatty acids and conjugated linoleic acid isomers of beef cattle, with emphasis on their nutritional value and discriminatory ability. Food Chem..

[46] Lourenço M., Van Ranst G., Vlaeminck B., De Smet S., Fievez V. (2008). Influence of different dietary forages on the fatty acid composition of rumen digesta as well as ruminant meat and milk. Anim. Feed Sci. Technol..

[47] Toral P.G., Monahan F.J., Hervas G., Frutos P., Moloney A.P. (2018). Review: Modulating ruminal lipid metabolism to improve the fatty acid composition of meat and milk. challenges and opportunities. Animal.

[48] Khiaosa-Ard R., Bryner S.F., Scheeder M.R.L., Wettstein H.-R., Leiber F., Kreuzer M., Soliva C.R. (2009). Evidence for the inhibition of the terminal step of ruminal α-linolenic acid biohydrogenation by condensed tannins. J. Dairy Sci..

[49] Campidonico L., Toral P.G., Priolo A., Luciano G., Valenti B., Hervás G., Frutos P., Copani G., Ginane C., Niderkorn V. (2016). Fatty acid composition of ruminal digesta and longissimus muscle from lambs fed silage mixtures including red clover, sainfoin, and timothy. J. Anim. Sci..

[50] Tava A., Avato P. (2006). Chemical and biological activity of triterpene saponins from medicago species. Nat. Prod. Commun..

[51] Dannenberger D., Nuernberg K., Nuernberg G., Scollan N., Steinhart H., Ender K. (2005). Effect of pasture vs. concentrate diet on CLA isomer distribution in different tissue lipids of beef cattle. Lipids.

[52] Lorenz S., Buettner A., Ender K., Nürnberg G., Papstein H.J., Schieberle P., Nürnberg K. (2002). Influence of keeping system on the fatty acid composition in the longissimus muscle of bulls and odorants formed after pressure-cooking. Eur. Food Res. Technol..

[53] Steen R.W.J., Lavery N.P., Kilpatrick D.J., Porter M.G. (2003). Effects of pasture and high-concentrate diets on the performance of beef cattle, carcass composition at equal growth rates, and the fatty acid composition of beef. N. Z. J. Agric. Res..

[54] Aboujaoude C., Pereira A.S.C., Feitosa F.L.B., Antunes De Lemos M.V., Chiaia H.L.J., Berton M.P., Peripolli E., Silva R.M.D.O., Ferrinho A.M., Mueller L.F. (2018). Genetic parameters for fatty acids in intramuscular fat from feedlot-finished Nelore carcasses. Anim. Prod. Sci..

[55] Leal-Gutiérrez J.D., Mateescu R.G. (2019). Genetic basis of improving the palatability of beef cattle: Current insights. Food Biotechnol..

[56] Joseph S.J., Robbins K.R., Pavan E., Pratt S.L., Duckett S.K., Rekaya R. (2010). Effect of diet supplementation on the expression of bovine genes associated with fatty acid synthesis and metabolism. Bioinform. Biol. Insights.

[57] De Smet S., Raes K., Demeyer D. (2004). Meat fatty acid composition as affected by fatness and genetic factors: A review. Anim. Res..

[58] French P., Stanton C., Lawless F., O’Riordan E.G., Monahan F.J., Caffrey P.J., Moloney A.P. (2000). Fatty acid composition, including conjugated linoleic acid, of intramuscular fat from steers offered grazed grass, grass silage, or concentrate-based diets. J. Anim. Sci..

[59] Aldai N., Dugan M.E.R., Kramer J.K.G., Martínez A., López-Campos O., Mantecón A.R., Osoro K. (2011). Length of concentrate finishing affects the fatty acid composition of grass-fed and genetically lean beef: An emphasis on trans-18:1 and conjugated linoleic acid profiles. Animal.

[60] McAfee A.J., McSorley E.M., Cuskelly G.J., Moss B.W., Wallace J.M.W., Bonham M.P., Fearon A.M. (2010). Red meat consumption: An overview of the risks and benefits. Meat Sci..

[61] Nuernberg K., Dannenberger D., Nuernberg G., Ender K., Voigt J., Scollan N.D., Wood J.D., Nute G.R., Richardson R.I. (2005). Effect of a grass-based and a concentrate feeding system on meat quality characteristics and fatty acid composition of longissimus muscle in different cattle breeds. Livest. Prod. Sci..

[62] Tansawat R., Maughan C.A.J., Ward R.E., Martini S., Cornforth D.P. (2013). Chemical characterisation of pasture- and grain-fed beef related to meat quality and flavour attributes. Int. J. Food Sci. Technol..

[63] Raes K., De Smet S., Demeyer D. (2004). Effect of dietary fatty acids on incorporation of long chain polyunsaturated fatty acids and conjugated linoleic acid in lamb, beef and pork meat: A review. Anim. Feed Sci. Technol..

[64] Enser M., Hallett K.G., Hewett B., Fursey G.A.J., Wood J.D., Harrington G. (1998). Fatty acid content and composition of UK beef and lamb muscle in relation to production system and implications for human nutrition. Meat Sci..

[65] Nuernberg K., Nuernberg G., Ender K., Lorenz S., Winkler K., Rickert R., Steinhart H. (2002). N-3 fatty acids and conjugated linoleic acids of longissimus muscle in beef cattle. Eur. J. Lipid Sci. Technol..

[66] Lee J.H., Min B.R. (2021). Carcass characteristics and meat quality of Kiko crossbred male goats as influenced by feeding phytochemical tannin containing supplementations. Agric. Sci..

[67] Zheng Y., Wang S., Yan P. (2018). The meat quality, muscle fiber characteristics and fatty acid profile in Jinjiang and F_1_ Simmental × Jinjiang yellow cattle. Asian-Australas. J. Anim. Sci..

[68] Cater N.B., Denke M.A. (2001). Behenic acid is a cholesterol-raising saturated fatty acid in humans. Am. J. Clin. Nutr..

[69] Klein C.M., Jenkins T.C. (2011). Docosahexaenoic acid elevates trans-18:1 isomers but is not directly converted into trans-18:1 isomers in ruminal batch cultures. J. Dairy Sci..

[70] Sampath H., Ntambi J.M. (2005). The fate and intermediary metabolism of stearic acid. Lipids.

[71] Pewan S.B., Otto J.R., Kinobe R.T., Adegboye O.A., Malau-Aduli A.E.O. (2021). Nutritional enhancement of health beneficial omega-3 long-chain polyunsaturated fatty acids in the muscle, liver, kidney, and heart of Tattykeel Australian white MARGRA lambs fed pellets fortified with omega-3 oil in a feedlot system. Biology (Basel).

[72] Moreira D.K.T., Santos P.S., Gambero A., Macedo G.A. (2017). Evaluation of structured lipids with behenic acid in the prevention of obesity. Food Res. Int..

[73] Maughan B., Provenza F.D., Tansawat R., Maughan C., Martini S., Ward R., Clemensen A., Song X., Cornforth D., Villalba J.J. (2014). Importance of grass-legume choices on cattle grazing behavior, performance, and meat characteristics. J. Anim. Sci..

[74] Blanco M., Joy M., Panea B., Albert P., Ripoll G., Carrasco S., Revilla R., Casas I. (2012). Effects of the forage content of the winter diet on the growth performance and carcass quality of steers finished on mountain pasture with a barley supplement. Anim. Prod. Sci..

[75] Monteiro A.C.G., Navas D.R., Lemos J.P.C. (2014). Effects of castration and time-on-feed on Mertolenga breed beef quality. Animal.

[76] De Brito G.F., McGrath S.R., Holman B.W.B., Friend M.A., Fowler S.M., van de Ven R.J., Hopkins D.L. (2016). The effect of forage type on lamb carcass traits, meat quality and sensory traits. Meat Sci..

[77] Ladeira M.M., Schoonmaker J.P., Swanson K.C., Duckett S.K., Gionbelli M.P., Rodrigues L.M., Teixeira P.D. (2018). Review: Nutrigenomics of marbling and fatty acid profile in ruminant meat. Animal.

[78] National Health and Medical Research Council (2013). Australian Code of Practice for the Care and Use of Animals for Scientific Purposes.

[79] Poppi D.P., Quigley S.P., Silva T.A.C.C., McLennan S.R. (2018). Challenges of beef cattle production from tropical pastures. Rev. Bras. Zootec..

[80] Agriculture and Resource Management Council of Australia and New Zealand (2001). Model Code of Practice for the Welfare of Animals: Livestock at Slaughtering Establishments.

[81] Commonwealth Scientific and Industrial Research Organisation (2007). Nutrient Requirements of Domesticated Ruminants.

[82] Malau-Aduli A.E.O., Siebert B.D., Bottema C.D.K., Pitchford W.S. (1998). Breed comparison of the fatty acid composition of muscle phospholipids in Jersey and Limousin cattle. J. Anim. Sci..

[83] Folch J., Lees M., Stanley G.H.S. (1957). A simple method for the isolation and purification of total lipids from animal tissues. J. Biol. Chem..

[84] Flakemore R.A., Balogun R.O., McEvoy P.D., Malau-Aduli B.S., Nichols P.D., Malau-Aduli A.E.O. (2014). Genetic variation in intramuscular fat of prime lambs supplemented with varying concentrations of degummed crude canola oil. Int. J. Nutr. Food Sci..

[85] AOCS—American Oil Chemists’ Society (2017). Slip Melting Point ISO Standard.

[86] Pewan S.B., Otto J.R., Kinobe R.T., Adegboye O.A., Malau-Aduli A.E.O. (2020). MARGRA lamb eating quality and human health-promoting omega-3 long-chain polyunsaturated fatty acid profiles of Tattykeel Australian white sheep: Linebreeding and gender effects. Antioxidants.

[87] Malau-Aduli A.E.O., Holman B.W.B., Kashani A., Nichols P.D. (2016). Sire breed and sex effects on the fatty acid composition and content of heart, kidney, liver, adipose and muscle tissues of purebred and first-cross prime lambs. Anim. Prod. Sci..

[88] Bligh E.G., Dyer W.J. (1959). A rapid method of total lipid extraction and purification. Can. J. Biochem. Physiol..

[89] Miller M.R., Nichols P.D., Barnes J., Davies N.W., Peacock E.J., Carter C.G. (2006). Regiospecificity profiles of storage and membrane lipids from the gill and muscle tissue of Atlantic salmon (*Salmo salar* L.) grown at elevated temperature. Lipids.

[90] Flakemore A.R., Malau-Aduli B.S., Nichols P.D., Malau-Aduli A.E.O. (2017). Omega-3 fatty acids, nutrient retention values, and sensory meat eating quality in cooked and raw Australian lamb. Meat Sci..

[91] Clayton E.H., Nicholls T.N.C. (2014). Graham Centre Monograph No. 4: Long-Chain Omega-3 Polyunsaturated Fatty Acids in Ruminant Nutrition: Benefits to Animals and Humans.

